# Testing for terrestrial and freshwater microalgae productivity under elevated CO_2_ conditions and nutrient limitation

**DOI:** 10.1186/s12870-023-04042-z

**Published:** 2023-01-13

**Authors:** Anastasiia Kryvenda, Rudolf Tischner, Bastian Steudel, Carola Griehl, Robert Armon, Thomas Friedl

**Affiliations:** 1 Albrecht-von-Haller-Institute for Plant Sciences, Department of Experimental Phycology and Culture Collection of Algae (SAG), University of Göttingen, Nikolausberger Weg 18, 37073 Göttingen, Germany; 2 Present address: Staatliche Betriebsgesellschaft für Umwelt und Landwirtschaft, 01683 Nossen, Germany; 3 Present address: Department of Health and Environmental Sciences, Xi’an Jiaotong-Liverpool University (XJTLU), Suzhou, 215123 Jiangsu Province China; 4 Department of Applied Biosciences and Process Technology, Competence Center Algal Biotechnology, Anhalt University of Applied Sciences, 06366 Köthen, Germany; 5 Technion-Israel Institute of Technology, Faculty of Civil and Environmental Engineering, 32000 Haifa, Israel

**Keywords:** Algae, Chlorophyceae, Trebouxiophyceae, Carbon dioxide, Growth, Biomass, Fatty acids, Carotenoids

## Abstract

**Background:**

Microalgae CO_2_ fixation results in the production of biomass rich in high-valuable products, such as fatty acids and carotenoids. Enhanced productivity of valuable compounds can be achieved through the microalgae’s ability to capture CO_2_ efficiently from sources of high CO_2_ contents, but it depends on the species. Culture collections of microalgae offer a wide variety of defined strains. However, an inadequate understanding of which groups of microalgae and from which habitats they originate offer high productivity under increased CO_2_ concentrations hampers exploiting microalgae as a sustainable source in the bioeconomy.

**Results:**

A large variety of 81 defined algal strains, including new green algal isolates from various terrestrial environments, were studied for their growth under atmospheres with CO_2_ levels of 5–25% in air. They were from a pool of 200 strains that had been pre-selected for phylogenetic diversity and high productivity under ambient CO_2_. Green algae from terrestrial environments exhibited enhanced growth up to 25% CO_2_. In contrast, in unicellular red algae and stramenopile algae, which originated through the endosymbiotic uptake of a red algal cell, growth at CO_2_ concentrations above 5% was suppressed. While terrestrial stramenopile algae generally tolerated such CO_2_ concentrations, their counterparts from marine phytoplankton did not. The tests of four new strains in liquid culture revealed enhanced biomass and chlorophyll production under elevated CO_2_ levels. The 15% CO_2_ aeration increased their total carotenoid and fatty acid contents, which were further stimulated when combined with the starvation of macronutrients, i.e., less with phosphate and more with nitrogen-depleted culture media.

**Conclusion:**

Green algae originating from terrestrial environments, Chlorophyceae and Trebouxiophyceae, exhibit enhanced productivity of carotenoids and fatty acids under elevated CO_2_ concentrations. This ability supports the economic and sustainable production of valuable compounds from these microalgae using inexpensive sources of high CO_2_ concentrations, such as industrial exhaust fumes.

**Supplementary Information:**

The online version contains supplementary material available at 10.1186/s12870-023-04042-z.

## Background

Microalgae, being able to store energy from sunlight and fundamental in the global carbon cycle, have attracted worldwide attention in biotechnology. Microalgae CO_2_ fixation is accompanied by the production of biomass which can be transformed into a great variety of high-value products, such as polyunsaturated fatty acids and carotenoids, e.g., [1–6]. Microalgae are accepted as a significant alternative source for renewable fuels [7, 8] and biogas [9]. They can also be efficiently employed in bioremediation processes, such as wastewater treatment and greenhouse gas mitigation [3]. Among the many advantages of microalgae is their high photosynthetic efficiency, resulting in fast growth and increased productivity [10]. The ability to tolerate high CO_2_ contents (5 to 15% and even higher) allows microalgae to capture CO_2_ efficiently from streams such as flue and flaring gases [1, 3]. Still, it is dependent on the species of microalgae [1, 7]. The concentration of CO_2_ in power plant exhaust fumes may vary between 6 and 13%, depending on the fuel composition [11]. In addition, or as an alternative to chemical and physical approaches, microalgae growth can considerably mitigate the CO_2_ contents of exhaust fumes [12, 13]. Approaches aiming at biofuel production also require algae strains, which produce biomass under high carbon dioxide concentrations [4, 14].

In the emerging field of microalgae-based processes and products, culture collections of microalgae are important resource centers providing a wide variety of defined algal strains. Their defined culture strains meet the high-quality standards required for bioeconomy due to their purity and genetic stability. Their constant availability from culture collections can ensure reproducibility (e.g., [15, 16]). However, only culture strains that have proven their agitation resistance and high productivity in liquid culture, preferably with simple, inexpensive growth media, appear suitable for the economic growth in industrial photobioreactors. Using flue gas can accelerate microalgal growth rates, boost biomass productivity, and thus increase the economic feasibility of the production of biomass and valuable compounds from microalgae [1–4]. However, though CO_2_ is the substrate of photosynthetic carbon fixation, higher CO_2_ concentration always results in the transient inhibition of photosynthesis and growth of algal cells [1, 17]. Therefore, it is important to find algal strains that are productive under elevated levels of CO_2_ in the air [3]. They offer industrial exhaust fumes to be used as an inexpensive source of CO_2_ and for agitating the algal suspension in photobioreactors [1, 4, 14].

Screenings for algal strains tolerating elevated CO_2_ concentrations have already been performed (e.g., [18, 19]). Almost all previous studies have used either small numbers of strains or a small phylogenetic diversity of algal strains. The experimental conditions used ranked from ambient air up to 20% CO_2_ in air while temperature, culture media, and illumination were varied. A mixed biodiverse microalgae community has been exposed to flue gas and *Desmodesmus* spp. (Sphaeropleales, Chlorophyceae) were the main surviving species after several months [4, 14]. A screening of 12 microalgal strains was performed at 2% CO_2_ in air, and green algae of the Chlorophyceae, i.e., *Chlamydomonas* spp. (Volvocales) and *Tetradesmus obliquus* (Sphaeropleales), found most suitable for biodiesel production [14]. The lipid production of the green algae *Botrycoccus braunii*, *Chlorella vulgaris* (both Trebouxiophyceae), and *Scenedesmus* spp. (Sphaeropleales, Chlorophyceae) under 10% CO_2_ revealed species-specific lipid production [20].

The aim of our study was a large-scale screening of a broad taxonomic breadth of defined algal strains from the SAG culture collection and evaluating them for their growth in high CO_2_ conditions. Those strains should be identified that can sustain or even exhibit positive responses to higher CO_2_ concentrations. The SAG culture collection has provided the public with pure, defined culture material for almost 70 years. Therefore, expertise has accumulated that microalgal strains isolated from various terrestrial habitats (e.g., soil and rock surfaces) are more robust than those from phytoplankton, i.e., they are sufficiently agitation-resistant and productive in simple liquid mineral culture media when bubbling with air at ambient CO_2_. In searching for robust and productive algal strains suited for the photobioreactor technology [21] under elevated levels of CO_2_ in the air, we isolated 12 new strains. Their origins included a range of habitats, i.e., biological soil crusts of a semi-desert and surfaces of sandy and mud soils to temporary shallow freshwaters (Table 1). Such environments may promote the growth of particularly robust microalgal species. In addition, we performed a screening of defined and already available microalgae strains from the SAG culture collection, representing different classes of algae. We tested selected examples from the Cyanobacteria, Rhodophyta, and stramenopile algae (Eustigmatophyceae, Xanthophyceae, and the diatom *Phaeodactylum*). All tested algal strains are quickly and constantly accessible from the SAG culture collection [15] or other culture collections (Table 2). The algal strains were maintained on agar plates exposed to atmospheres of 5–25% CO_2_ in air, which allowed for defined conditions independent of the CO_2_ gas dissolution in the liquid phase [22]. Four strains from the new terrestrial isolates were most promising because of their CO_2_ tolerance and productivity. Therefore, we selected them for further growth experiments with CO_2_ aeration in liquid culture as required for photobioreactor technology. We explored their production of valuable compounds, i.e., carotenoids and fatty acids, under the gassing of air with elevated CO_2_. Finally, the selected strains were subjected to starvation in macronutrients, i.e., phosphorous and nitrogen, to test whether this could further stimulate valuable compound productivity in combination with elevated CO_2_ concentrations.

**Table 1:** The 12 newly isolated strains, their species identification, origins, sequence accessions, sequence identities with closest reference sequences, and their growth pattern under elevated CO_2_ atmospheres

species identification	Strain	isolation source; latitude, longitude	sequence accession no.	length ITS2	sequence identity: accession no. of closest reference sequence	growth pattern
*Chlamydomonas* sp.	SAG 2630	Temporary freshwater pond with algal bloom (Israel, Haifa); 32.808119N, 35.020541E	MZ546610	235	98%: MH311547	2
*Chlorella vulgaris*	SAG 2629	Temporary freshwater rivulet, iron-rich (Germany, Bad Pyrmont); 51.988757N, 9.252696E	MZ546604	244	100%: AY591499, and 94 other	4
*Chlorella vulgaris*	SAG 2606	Temporary freshwater rivulet, iron-rich (Germany, Bad Pyrmont); 51.988757N, 9.252696E	MZ546608	244	100%: AY591499, and 94 other	5
*Desmodesmus armatus*	SAG 2635	Soil surface of a meadow (Ukraine); 46.480278N, 33.849722E	MZ546611	245	100%: MK975484, and 12 other	7
*Desmodesmus multivariabilis*	SAG 2628	Biofilm on soil surface (Germany, Uslar); 51.649100N, 9.750686E	MZ546603	248	100%: MH311545	7
*Pseudomuriella aurantiaca*	SAG 2631	Biofilm on soil surface (Israel, Haifa); 32.778451N, 35.025604E	MZ546609	247	99%: MH703741, and 2 other	5
*Tetradesmus arenicola*	SAG 2632	Biological soil crust on the surface of sandy soil (Ukraine); 46.576798N, 31.512473E	MZ546602	240	100% MH703775, and 3 other	4
*Tetradesmus arenicola*	SAG 2633	Surface of sandy soil (Ukraine)	MZ546612	240	100%: MH703775, and 3 other	2
*Tetradesmus bajacalifornicus*	BIOTA 136	Biological soil crust on the surface of sandy soil (South Africa, semi-desert); 30.1865S, 17.5433E	MZ546605	243	95%: HQ246450, and 4 other	7
*Tetradesmus deserticola*	BIOTA 153	Biological soil crust on the surface of sandy soil (South Africa, semi-desert); 30.3856S, 18.2757E	ON677848	240	98%: AY510471	2
*Tetradesmus obliquus*	SAG 2607	Biofilm on soil surface next to a freshwater pond (Germany, Uslar); 51.647250N, 9.761198E	MZ546607	240	100%: MK975482, and 69 other	6
*Tetradesmus obliquus*	SAG 2608	Biofilm on soil surface next to a freshwater pond (Germany, Uslar); 51.647250N, 9.761198E	MZ546606	240	100%: MK975482, and 69 other	6

## Results

### Identification of the new strains

Sequence comparisons of the ITS2 rDNA revealed the 12 new green algal strains to share high similarities, i.e., 95–100%, with available references (Table 1). This identified the strains as four different species of *Tetradesmus* (*T. arenicola*, *T. bajacalifornicus, T. deserticola*, and *T. obliquus*), two species of *Desmodesmus* (*D. armatus* and *D. multivariabilis*), *Pseudomuriella aurantiaca*, and *Chlorella vulgaris*. The strain SAG 2630 shared high sequence similarity (98%) with an unidentified *Chlamydomonas* sp. (Volvocales, Chlorophyceae) and, therefore, was left unidentified at the species level.

### Preselection of test strains, evaluation of their growth under elevated CO_2_ atmospheres

About 200 strains from the SAG culture collection, and one strain from the Culture Collection of Algae and Protozoa (CCAP; www.ccap.ac.uk), were examined for their growth properties. Of those, 69 strains appeared promising for testing their growth under atmospheres of elevated CO_2_ levels. Finally, 81 strains were tested for their growth properties under atmospheres of elevated CO_2_ levels in air (Table 2; Additional file 1: Fig. S1). They were represented by green algae (Chlorophyta), i.e., the classes Chlorophyceae (29 strains), Trebouxiophyceae (17 strains), and Chlorodendrophyceae (1 strain), unicellular red algae (Rhodophyta; 13 strains), stramenopile algae, i.e., classes Eustigmatophyceae (7 strains), Xanthophyceae (5 strains), and the diatom *Phaeodactylum tricornutum* (3 strains), and Cyanobacteria (6 strains).

**Table 2:** The 7 growth patterns and their distribution over the 81 tested strains

growth pattern	class - phylum	species and strain	no. of strains
**7**	Chlorophyceae - Chlorophyta	*Desmodesmus armatus* SAG 2635^1^, *D. komarekii* CCAP 258/232, *D. multivariabilis* SAG 2628^1^, *Tetradesmus bajacalifornicus* BIOTA 136^1^	4
**6**	Chlorophyceae - Chlorophyta	*Tetradesmus almeriensis* SAG 2066, *T. obliquus* SAG 276-3b, *T. obliquus* SAG 276–6, *T. obliquus* SAG 2607^1^, *T. obliquus* SAG 2608^1^	5
**5**	Chlorophyceae - Chlorophyta	*Bracteacoccus giganteus* SAG 2272, *B. minor* SAG 61.80, *Cylindrocapsa involuta* SAG 314–1, *Haematococcus pluvialis* SAG 192.80, *H. pluvialis* SAG 34 − 1a, *H. pluvialis* SAG 34 − 1b, *Pseudomuriella aurantiaca* SAG 2631^1^, *Spongiochloris* sp. SAG 2515	8
	Trebouxiophyceae - Chlorophyta	*Chlorella sorokiniana* SAG 211–34, *C. vulgaris* SAG 211-11b, *C. vulgaris* SAG 2606^1^, *C. vulgaris* SAG 9.88, *Deuterostichococcus epilithicus* SAG 2060, *Micractinium simplicissimum* SAG 15.93, *Myrmecia israeliensis* SAG 2228, *Stichococcus bacillaris* SAG 2118	8
	Eustigmatophyceae - Stramenopiles	*Vischeria polyphem* SAG 38.84	1
	Xanthophyceae - Stramenopiles	*Heterococcus viridis* SAG 2422	1
	Bacillariophyceae *-* Stramenopiles	*Phaeodactylum tricornutum* SAG 1090–6	1
**4**	Chlorodendrophyceae - Chlorophyta	*Tetraselmis tetrahele* SAG 161 − 2c	1
	Chlorophyceae - Chlorophyta	*Haematococcus pluvialis* SAG 44.96, *H. pluvialis* SAG 49.94, *Spongiochloris* sp. SAG 2514, *Tetradesmus arenicola* SAG 2632^1^	4
	Trebouxiophyceae - Chlorophyta	*Chlorella vulgaris* SAG 2629^1^	1
**3**	Cyanophyceae - Cyanobacteria	*Fischerella muscicola* SAG 1427–1, *Micrococleus autumnalis* SAG 35.90, *Nostoc commune* SAG 1453–3	3
	Porphyridiophyceae - Rhodophyta	*Porphyridium purpureum* SAG 112.79, *P. purpureum* SAG 113.79, *P. purpureum* SAG 1380 − 1e, *P. sordidum* SAG 114.79, *P. sordidum* SAG 2356	5
	Rhodellophyceae - Rhodophyta	*Dixoniella grisea* SAG 72.90	1
	Chlorophyceae - Chlorophyta	*Ettlia carotinosa* SAG 213–4, *Neochlorosarcina negevensis* SAG 67.80, *Radiosphera negevensis* SAG 87.80	3
	Trebouxiophyceae - Chlorophyta	*Neocystis brevis* SAG 40.86, *Viridiella fridericiana* SAG 10.92	2
	Eustigmatophyceae - Stramenopiles	*Vischeria helvetica* SAG 876–1, *V. magna* SAG 36.89, *V. stellata* SAG 887–2	3
	Xanthophyceae - Stramenopiles	*Ophiocytium parvulum* SAG 37.84	1
**2**	Cyanophyceae - Cyanobacteria	*Nostoc microscopicum* SAG 55.79	1
	Porphyridiophyceae - Rhodophyta	*Porphyridium purpureum* SAG 1380 − 1a, *P. purpureum* SAG 1380 − 1b, *P. purpureum* SAG 1380 − 1c, *P. purpureum* SAG 1380 − 1d, *P. purpureum* SAG 1380 − 1f, *P. sordidum* SAG 44.94	6
	Rhodellophyceae - Rhodophyta	*Rhodella violacea* SAG 115.79	1
	Chlorophyceae - Chlorophyta	*Chlamydomonas* sp. SAG 2630^1^, *Chlorococcum novae-angeliae* SAG 5.85, *Haematococcus* sp. SAG 2122, *Tetradesmus arenicola* SAG 2633^1^, *T. deserticola* BIOTA 153^1^	5
	Trebouxiophyceae - Chlorophyta	*Chloroidium angusto-ellipsoideum* SAG 2041, *Coccomyxa avernensis* SAG 216–1, *Lobosphaera incisa* SAG 2007, *Stichococcus ampulliformis* SAG 2047, *S. bacillaris* SAG 335–3, *S. bacillaris* SAG 379 − 1d	6
	Eustigmatophyceae - Stramenopiles	*Nannochloropsis oculata* SAG 38.85	1
	Xanthophyceae - Stramenopiles	*Heterococcus leptosiroides* SAG 2420, *H. leptosiroides* SAG 2425	2
	Bacillariophyceae *-* Stramenopiles	*Phaeodactylum tricornutum* SAG 1090 − 1a, *P. tricornutum* SAG 1090 − 1b	2
**1**	Cyanophyceae - Cyanobacteria	*Gloeothece aequatorialis* SAG 36.87, *Nostochopsis lobatus* SAG 2.97	2
	Eustigmatophyceae - Stramenopiles	*Microchloropsis gaditana* SAG 2.99, *M. salina* SAG 40.85	2
	Xanthophyceae - Stramenopiles	*Heterococcus viridis* SAG 2424	1

^1^newly isolated strain

**Fig. 1:**
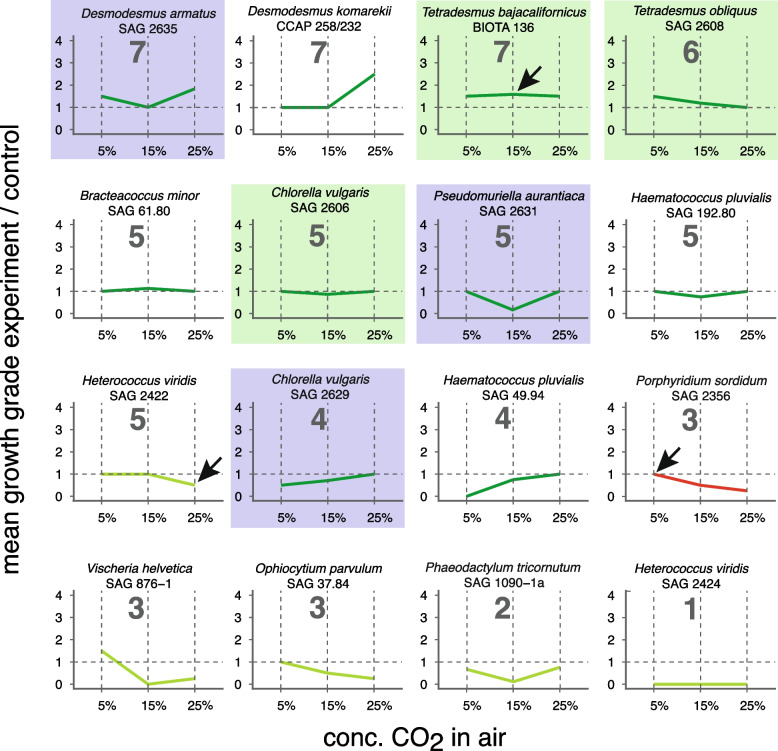
Example diagrams showing the 7 patterns revealed for the growth of algal strains on solid media under atmospheres of elevated CO_2_ concentrations in air. Bold numbers, general patterns of mean growth (see text). The graph shows the mean growth from 3 replicate experiments in relation to controls under ambient CO_2_. Arrows mark examples for the different relations, i.e., 1, no change; > 1, enhanced growth; < 1 decreased growth; blue, example diagrams for the new terrestrial green algal isolates (see Table 1); green, examples diagrams for the 4 strains selected for further testing (see text)

For the visual assessment of algal growth 2 weeks after the start of the experiment, five grades were assigned. A “0” was given to no visible growth at all and bleached colonies, “0.5” for stagnant growth with pale colonies, and “1” for colonies with low growth still not spreading over the agar surface. Grade “2” was given to colonies of bright color scattered over the agar surface, and “3” to those of intense dark color and densely spreading over the agar surface (Additional file 1: Fig. S1). The ratio of the growth grade between CO_2_ treatment and that of ambient (control) was calculated for the three replicates, and their mean values were graphically displayed (Fig. 1, Additional file 2: Fig. S2). For example, a ratio of 0.5 (e.g., 1:2) meant an adverse effect of elevated CO_2_ with stagnant growth, and a ratio of 1 (e.g., 2:2) tolerance with no growth change under elevated CO_2_ with proper growth. A ratio of 1.5 (e.g, 3:2) meant accelerated growth due to elevated CO_2_ (Additional file 1: Fig. S1).

The screening of the 81 test strains revealed 7 distinct growth patterns based on their growth grade ratios under 5, 15, and 25% CO_2_ in air atmospheres (Fig. 1, Additional file 2: Fig. S2). Those with growth patterns 7 and 6 exhibited enhanced growth of their algal colonies under elevated CO_2_ concentrations in the air atmosphere. The strains with growth pattern 7 had enhanced growth under 25% CO_2_ (Fig. 1, Additional file 2: Fig. S2). It was observed in the green algae (Chlorophyta, Chlorophyceae), i.e., 2 strains of *Desmodesmus*, and *Tetradesmus bajacalifornicus* BIOTA 136 (Table 2). The latter strain showed enhanced growth at all tested levels of elevated CO_2_ atmospheres (Fig. 1). In *D. komarekii* CCAP 258/232, growth under the 5 and 15% CO_2_ atmospheres was unchanged (Fig. 1). Strains with growth pattern 6 had enhanced growth under the 5% CO_2_ atmosphere. Growth was (almost) unchanged under the other elevated levels (Fig. 1, Additional file 2: Fig. S2). Only 2 species of *Tetradesmus* exhibited pattern 6 (Table 2). In growth patterns 5, 4, and 3, there was no growth enhancement but tolerance towards elevated CO_2_ levels. In pattern 5, unchanged growth under two elevated levels, mostly 5, and 25% CO_2,_ was found. Growth decreased under the 15% CO_2_ atmosphere (Fig. 1, Additional file 2: Fig. S2). Pattern 5 was found to be rather common among the tested strains, i.e., in about one-third (16) strains of green algae (Chlorophyta) and 3 strains of stramenopile algae (Table 2). Among the latter *Vischeria polyphem* SAG 38.84 (Eustigmatophyceae) and *Heterococcus viridis* SAG 2422 (Xanthophyceae) differed from other strains with growth pattern 5 as they exhibited unchanged growth under the 5 and 15% CO_2_ atmospheres, but only under 25% CO_2_ their growth decreased (Fig. 1, Additional file 2: Fig. S2). Growth pattern 4 featured unchanged growth under the highest CO_2_ level tested, 25%. Growth was decreased under the 5 and 15% CO_2_ atmospheres in air (Fig. 1). Only 6 strains of green algae exhibited growth pattern 4 (Table 2). Growth pattern 3 was defined by unchanged growth under the 5% CO_2_ atmosphere and continuously decreasing growth under the higher concentrated CO_2_ atmospheres (Fig. 1, Additional file 2: Fig. S2). There were 18 tested strains with pattern 3. Thus, it was as common as pattern 5 but widely distributed over all tested algal lineages, including cyanobacteria (Table 1). *Vischeria helvetica* SAG 876–1 growth under the 5% CO_2_ atmosphere was slightly increased (Fig. 1). There were two growth patterns, 2 and 1, where all tested levels of increased CO_2_ concentrations in the air atmosphere had a negative impact on the algal growth. Strains exhibiting pattern 2 had reduced growth at all three tested levels (Fig. 1, Additional file 2: Fig. S2). It was the pattern most common and present in all tested algal lineages and cyanobacteria (Table 2). The strains of pattern 1 did not grow at all under the tested elevated CO_2_ levels in air atmospheres (Fig. 1, Additional file 2: Fig. S2). It was found in 2 strains of cyanobacteria and 3 strains of stramenopile algae (Table 2).

Out of the 12 new green algal strains (Table 1), only *Chlamydomonas* sp. SAG 2630, *Tetradesmus arenicola* SAG 2633, and *T. deserticola* BIOTA 153 were retarded in their growth under elevated CO_2_ concentrations. They exhibited growth pattern 2 (Table 1; Additional file 2: Fig. S2). All other new strains tolerated the 25% atmosphere (Additional file 2: Fig. S2). Remarkably, out of the 9 new green algal strains that were isolated from the surfaces of soils (Table 1), 3 strains were among those very few (4) that exhibited the best growth in high CO_2_ (pattern 7; Fig. 1). Those were *Desmodesmus armatus* SAG 2635, *D. multivariabilis* SAG 2628, and *Tetradesmus bajacalifornicus* BIOTA 136. They exhibited enhanced growth under the highest tested CO_2_ concentration, 25% (growth pattern 7; Table 1; Additional file 2: Fig. S2). The two new strains of *T. obliquus*, SAG 2607 and 2608, which were from the surface of moist soil, exhibited increased growth under the 5% and no growth change under the other elevated CO_2_ atmospheres (pattern 6; Additional file 2: Fig. S2).

Variations regarding the effects of elevated CO_2_ concentrations in the air atmosphere among different strains of the same species were noticed. The two new strains of *C. vulgaris* differed in their growth under the 5 and 15% CO_2_ atmospheres (growth patterns 4, 5) and those of *T. arenicola* under the 25% CO_2_ atmospheres (growth patterns 4 and 2; Additional file 2: Fig. S2). The strains of *C. vulgaris* and *T. arenicola* were isolated from closely neighboring locations, respectively (Table 1). Corresponding within-species differences were found for the red algae *Porphyridium purpureum* and *P. sordidum* (Porphyridiophyceae; growth patterns 3 and 2), and the green alga *Haematococcus pluvialis* (Chlorophyceae; growth patterns 5 and 4) (Additional file 2: Fig. S2). Among Xanthophyceae test strains, only one of the two *Heterococcus viridis* strains tolerated atmospheres of elevated CO_2_ (growth patterns 5 and 1; Additional file 2: Fig. S2).

For all tested Cyanobacteria strains, CO_2_ atmospheres higher than 5% resulted in suppressed growth (growth patterns 3 and 2, Table 2). Two cyanobacteria strains did not grow, even under the 5% CO_2_ atmosphere (growth pattern 1, Table 2). In about half of the tested strains representing unicellular red algae (Rhodophyta), 5% was the only level of elevated CO_2_ tolerated. In contrast, all higher CO_2_ levels led to suppressed growth (growth patterns 3 and 2, Table 2). However, just for one single strain, *Porphyridium purpureum* SAG 1380-1a, the 25% CO_2_ atmosphere did not affect its growth. Among strains of stramenopile algae, class Eustigmatophyceae, the strains from terrestrial habitats of the genus *Vischeria* tolerated 5% CO_2_, with *V. polyphem* strain SAG 38.84 even 15% CO_2_ in the atmosphere (growth patterns 3 and 5, Table 2). However, the growth of the tested strains from marine phytoplankton, the genera *Microchloropsis* and *Nannochloropsis*, was suppressed, or it even ceased at all elevated CO_2_ levels (growth patterns 2 and 1, Table 2). It confirms earlier studies on the CO_2_ utilization of *N. oculata* in response to CO_2_ aeration [23]. From the tested stramenopile algal strains of the class Xanthophyceae, only a single strain of a typical terrestrial (soil) alga, *Heterococcus viridis* SAG 2422, was left unaffected in growth until 15% CO_2_ in the atmosphere. However, *Ophiocytium parvulum* strain SAG 37.84 from an aquatic environment tolerated only the 5% CO_2_ atmosphere. The other tested Xanthophyceae strain reacted with suppressed or even ceased growth to elevated CO_2_ levels in the atmosphere (growth patterns 5, 3, 2, and 1, Table 2). Among the three tested strains of the diatom *Phaeodactylum tricornutum*, a diatom isolated from marine or brackish environments, the growth of two strains was suppressed under all tested CO_2_ levels (growth patterns 4 and 2, Table 2). Only one strain, SAG 1090–6, did not exhibit adverse effects on the growth under the 5 and 25% atmospheres.

### Biomass, chlorophyll, total carotenoid, and fatty acid contents of 4 selected green algal isolates in liquid culture

**Fig. 2:**
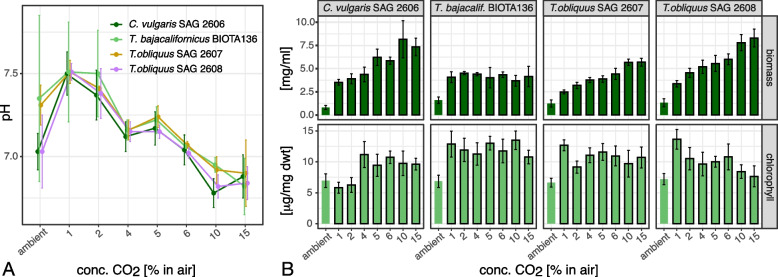
Growth experiments with 4 selected green algal strains in lquid cultures with aeration of increasing CO_2_ concentrations (ambient to 15%). Experiments were performed in quadruplicate, data expressed as mean ± SD. **A** Changes in the pH of liquid culture media measured at the end of a ten-day cultivation period. An equilibrium in the range of the CO_2_ buffer system was reached at the highest CO_2_ concentration for all four strains. **B** Biomass (dark green) and chlorophyll (light green) productivity at the end of a ten-day cultivation period

We selected four new green algal isolates as examples to further test terrestrial microalgae (including one from a temporary freshwater rivulet) for their productivity of biomass and total carotenoid contents. To resemble processes in photobioreactors, we performed the tests in liquid culture, i.e., liquid culture medium that was aerated with CO_2_ at either ambient or 15% concentration. One selected strain was *Tetradesmus bajacalifornicus* SAG BIOTA 136, the only strain exhibiting enhanced growth under all elevated CO_2_ atmospheres (growth pattern 7; Fig. 1). Two more strains were *T. obliquus* SAG 2607 and SAG 2608, which differed in their tolerance towards 15% CO_2_, i.e., with growth unaffected (SAG 2607) or slightly enhanced (SAG 2608) (growth pattern 6; Fig. 1, Additional file 2: Fig. S2). Finally, *Chlorella vulgaris* strain SAG 2606 represented those strains tolerating all three tested CO_2_ levels without change in growth (growth pattern 5; Fig. 1, Additional file 2: Fig. S2), one of the most common growth patterns among the tested green algal strains (Table 2). A prerequisite for the tests in liquid culture was to ensure that the found effects would concern just those caused by the enhanced (15%) CO_2_ concentration. There is the possibility that changes in the pH value generated by the CO_2_ aeration may interfere as a selection criterion. However, only a relatively small alkalization was observed due to the CO_2_ aeration from ambient to 15% CO_2_ (Fig. 2A). The consumption of CO_2_ and nitrate largely overcompensated the potential acidification. The metabolism of both leads to a slight alkalization of the medium. It is based on an H ^+^- cotransport (in the case of nitrate) or Na ^+^- cotransport (partly for CO_2_) via the plasma membrane [24]. Therefore, the pH remains within the buffer range of the carbonate buffer system, i.e., at pH 6.5 [22]. The observed effects are not the result of a pH change but are based on the high supply of CO_2_.

We analyzed the effects along with CO_2_ levels from ambient to 15% in the aeration on biomass productivity and chlorophyll (Fig. 2B). A continuous increase in biomass with increased CO_2_ supply was observed for *C. vulgaris* and the two *T. obliquus* strains. In contrast, *T. bajacalifornicus* achieved its highest biomass productivity at 2% CO_2,_ and there was no further increase with higher CO_2_ concentrations (Fig. 2B). The chlorophyll content in the three *Tetradesmus* strains (Fig. 2B) increased sharply at 1% CO_2_ to a level where almost no alterations occurred with further increasing CO_2_ concentrations. However, in *T. obliquus* SAG 2608, chlorophyll content decreased at 10 and 15% CO_2_, almost to the level under ambient CO_2_ concentration (Fig. 2B). In *C. vulgaris* SAG 2606, there was a similar sharp increase to a level at which further supply of CO_2_ had hardly any effect.

**Fig. 3:**
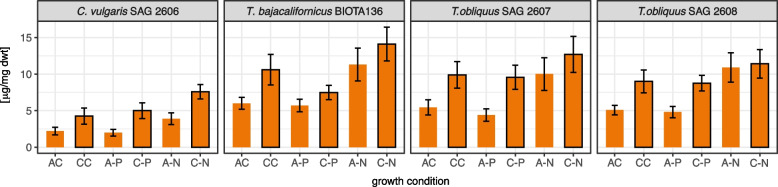
The contents of carotenoids of the four selected terrestrial new green algal strains in full medium or under limited macronutrient supply, at ambient or 15% CO_2_ aeration in liquid culture. Growth conditions: AC, complete liquid medium aerated with ambient CO_2_; CC, complete medium and aeration at 15% CO_2_; A-P and C-P, aeration with ambient and 15% CO_2_ at phosphate limitation; A-N and C-N, aeration with ambient and 15% CO_2_ at nitrogen limitation. Data are expressed as mean ± SD from experiments performed in quadruplicate

Total carotenoid contents in the four strains were examined at 15% CO_2_ aeration (condition CC) compared to the aeration at ambient CO_2_ (condition AC) in the complete liquid culture medium. In the three *Tetradesmus* strains, the total carotenoid contents under ambient CO_2_ (condition AC) were almost double as high or higher than in *C. vulgaris* SAG 2606 (Fig. 3). Aeration with 15% CO_2_ (condition CC) almost doubled the total carotenoid content in all four strains compared to ambient CO_2_ in air (condition AC). Carotenoid production may be a significant sink for the excess carbon under elevated CO_2_ supply in these strains. This may indicate different response strategies towards high CO_2_ levels in both green algal genera.

Nitrogen and phosphorus limitations were applied separately to test whether they further increased or decreased the effects of elevated CO_2_ on the four strains. In all four strains, nitrogen limitation at ambient CO_2_ (condition A-N) increased the total carotenoid content compared to that in the complete medium (condition AC; Fig. 3). Aeration with 15% CO_2_ under nitrogen limitation (condition C-N) further increased the carotenoid content (Fig. 3). Phosphate limitation at ambient CO_2_ (condition A-P) left the total carotenoid content almost unchanged compared to that in the complete medium (condition AC; Fig. 3). Elevated CO_2_ with phosphate limitation (condition C-P) doubled the total carotenoid content except for *T. bajacalifornicus* BIOTA 136 (Fig. 3). The latter exhibited only a slight increment (Fig. 3). Generally, the total carotenoid contents under phosphate limitation and elevated CO_2_ (condition C-P) were lower than under nitrogen limitation (condition C-N; Fig. 3).

**Fig. 4:**
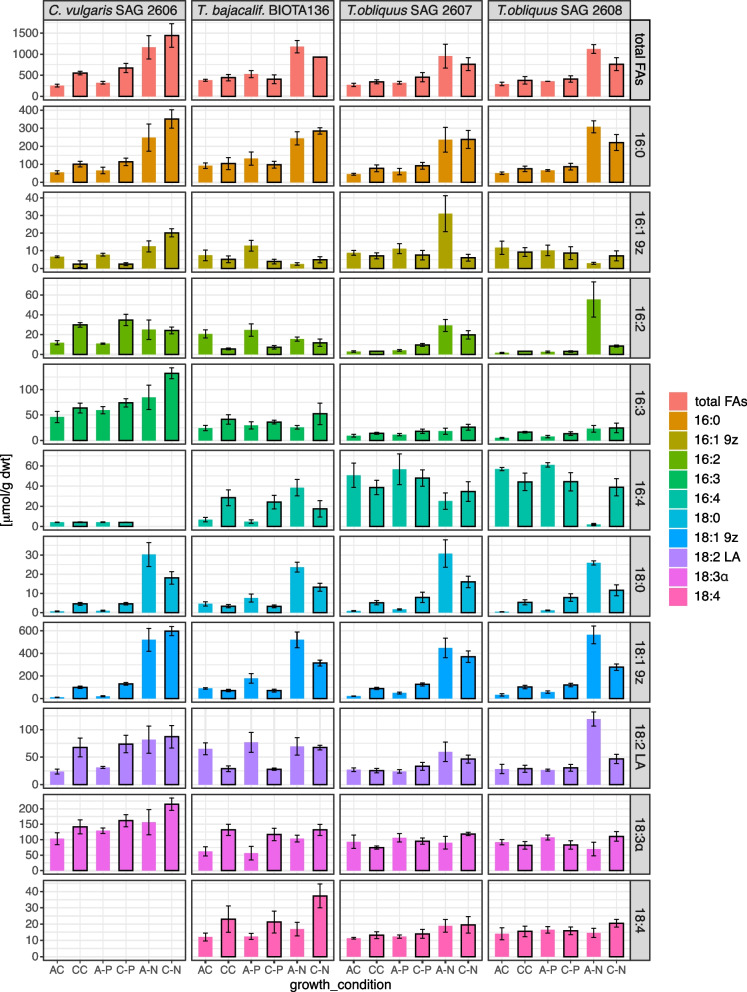
The contents of total fatty acids and 10 selected fatty acids in the four selected green algal strains in the complete or nutrient-limited growth medium, at ambient or 15% CO_2_ aeration in liquid culture. Growth conditions: AC, complete liquid medium aerated with ambient CO_2_; CC, complete medium and aeration at 15% CO_2_; A-P, C-P, aeration with ambient or 15% CO_2_ at phosphate limitation; A-N, C-N, aeration with ambient or 15% CO_2_ at nitrogen limitation. Data are expressed as mean ± SD from experiments performed in quadruplicate

The four green algal strains were also used to test their productivity of total fatty acids under elevated (15%) CO_2_ (condition CC). Under ambient CO_2_ (condition AC), the total fatty acid content was highest in *T. bajacalifornicus* compared to the other three strains (Fig. 4, top; Additional file 3: Table S1). Elevated CO_2_ (condition CC) increased the total fatty acid production in all four strains (Fig. 4). It was most pronounced in *C. vulgaris* SAG 2606*,* where the total fatty acid content almost doubled (Fig. 4, top; Additional file 3: Table S1). In the three *Tetradesmus* strains, the increase was only about 15–30% (Fig. 4, top; Additional file 3: Table S1).

We analyzed the CO_2_ effects on the contents of 10 selected fatty acids. They were polyunsaturated fatty acids (PUFAs; Fig. 4), except the 16:0 and 18:0 fatty acids. They were measured as fatty acid methyl esters (FAMEs). *C. vulgaris* SAG 2606 was distinct because it was the only tested strain where no 18:4 fatty acid was found (Fig. 4). Also, it exhibited the highest contents of the 18:3α and 16:3 fatty acids under the AC and CC conditions (Fig. 4). In *C. vulgaris* SAG 2606, elevated CO_2_ (condition CC) increased the contents of all fatty acids, except those of 16:1 9z and 16:3 (Fig. 4; Additional file 3: Table S1). In *Tetradesmus*, however, the contents of only some fatty acids increased under condition CC. It increased the contents of the 16:0, 16:3, and 18:4 fatty acids, whereas it decreased the 16:1 9z fatty acid contents compared to ambient CO_2_ (condition AC; Fig. 4; Additional file 3: Table S1). In strain *T. bajacalifornicus* BIOTA 136, the 16:4 and 18:3𝛼 fatty acid contents also increased, in contrast to *T. obliquus*. Also, its 18:4 fatty acid increment was more pronounced. However, its 18:2 LA fatty acid content considerably decreased compared to the corresponding contents of *T. obliquus* (Fig. 4; Additional file 3: Table S1).

We further tested whether nitrogen and phosphorus limitations could further increase or decrease the effects of elevated CO_2_ on fatty acids. N-limitation alone (condition A-N) stimulated all four strains to increase the total fatty acid content compared to condition AC (Fig. 4, top; Additional file 3: Table S1). It led to a robust increase in the 16:0, 18:0, and 18:1 9z fatty acid contents (Fig. 4). The increase was most pronounced for the 16:1 9z fatty acid in *Tetradesmus obliquus* SAG 2607 and for the 16:2, and 18:2 LA fatty acids in *T. obliquus* SAG 2608. In *C. vulgaris* SAG 2606, the 16:4 fatty acid, already low under condition AC, was not detectable under condition A-N (Fig. 4; Additional file 3: Table S1). Similarly, it reduced the 16:4 fatty acid contents in *T. obliquus* but substantially increased it in *T. bajacalifornicus* BIOTA 136 (Fig. 4; Additional file 3: Table S1). When combined with elevated CO_2_ (condition C-N), a further increase in the total fatty acid content compared to condition A-N was found only in *C. vulgaris* SAG 2602 (Fig. 4, top). Out of the 8 fatty acids detected in this strain under N-limitation, 7 fatty acids showed a further increase under the C-N condition. Only the 18:0 fatty acid content was reduced. In *Tetradesmus*, the 16:3, 18:3𝛼, and 18:4 fatty acid contents increased under the C-N condition. In contrast, contents of the 16:2, 18:0, and 18:1 9z fatty acids decreased compared to the A-N condition (Fig. 4; Additional file 3: Table S1). In *T. bajacalifornicus* BIOTA 136, the 18:4 fatty acid content more than doubled. Also, its 16:0 and 16:1 9z fatty acids contents increased, whereas its 16:4 fatty acid content decreased under the C-N condition (Fig. 4; Additional file 3: Table S1).

Phosphorus limitation under ambient CO_2_ (condition A-P) only slightly increased the total fatty acids compared to the complete medium (condition AC) in all tested strains (Fig. 4, top; Additional file 3: Table S1). In combination with elevated CO_2_ (condition C-P), total fatty acid contents increased in *C. vulgaris* and *T. obliquus*. However, it decreased in *T. bajacalifornicus* (Fig. 4, top; Additional file 3: Table S1). The contents of the selected 10 fatty acids under condition A-P were about the same as under condition AC (Fig. 4). The detailed analysis, however, showed a slight increase (Additional file 3: Table S1). Under condition C-P, the contents of fatty acids detected in *C. vulgaris* increased, except 16:1 9z fatty acid, which decreased (Fig. 4; Additional file 3: Table S1). In *T. obliquus,* condition C-P slightly increased the contents of the 16:0, 16:3, 18:0, and 18:2 LA fatty acids. The 16:4 fatty acid content decreased, and that of the 18:3𝛼 and 18:4 fatty acids stayed almost unchanged (Fig. 4; Additional file 3: Table S1). In *T. bajacalifornicus* BIOTA 136, however, the 16:4, 18:3𝛼, and 18:4 fatty acid contents considerably increased, whereas the other fatty acids stayed about the same or decreased compared to condition A-P (Fig. 4; Additional file 3: Table S1). We conclude that P-limitation had negligible effects on the fatty acid contents. In all strains, the N-limitation had the most significant impact on the contents of all the ten tested fatty acids (Fig. 4).

## Discussion

Our study has performed a large-scale screening of defined algal strains from the SAG culture collection and evaluated them for their growth in high CO_2_ conditions. The screening also included several new green algal strains from terrestrial and freshwater habitats. Our study aimed at identifying algal strains that can sustain or even exhibit positive responses to higher CO_2_ concentrations. Based on our findings, we suggest some of the screened strains be used for the sustainable production of valuable compounds, e.g., carotenoids and fatty acids, using inexpensive sources of CO_2_.

### Evaluation of growth experiments

Previous studies found that only relatively few strains can sustain growth under higher CO_2_ concentrations. Among those were several green algal strains of the Chlorophyceae, i.e., *Desmodesmus* and *Tetradesmus* (“*Scenedesmus*”) [1]. Our study expands the recovered phylogenetic breath of *Tetradesmus* species well suited for CO_2_ mitigation [1] by adding new strains of *T. bajacalifornicus* and *T. obliquus*. In previous studies, only 2 out of 74 (2.7%) and 17 out of 96 (17.7%) test strains were found tolerant [25, 26]. Among the test strains of our study, however, a total of 34 strains (39.5%) reacted positively with CO_2_ tolerance or even improved growth. Also, it is most likely because our strain selection covered a larger phylogenetic breadth. Particularly in the green algae, an unprecedented diversity of genera was tested. Our screening under elevated CO_2_ atmospheres on solid media revealed the green algae (Chlorophyta) as the best candidates for the sustainable production of carotenoids and fatty acids under high CO_2_ conditions. Identical culture conditions were applied to the 81 test strains for growing them on solid media under ambient and elevated CO_2_ concentrations. The solid culture media were the same that have been used to maintain the algal strains in the SAG culture collection since many years. Consequently, the observed differences among the growth experiments were due to genetic differences in strain or species.

Tolerance to elevated CO_2_ atmospheres but without enhanced growth (growth pattern 5) was found in most of the tested green algal strains from the Chlorophyceae and Trebouxiophyceae. The new isolate of *Chlorella vulgaris* SAG 2606, a member of Trebouxiophyceae, served as an example of green algae exhibiting growth pattern 5. In elevated CO_2_ aerated liquid cultures, *C. vulgaris* SAG 2606 performed with the best values in biomass production among the tested strains. It also exhibited the ability to increasingly accumulate carotenoids and fatty acids with higher CO_2_ concentrations. A particular advantage was that CO_2_, in combination with nitrogen limitation, enhanced the production of total fatty acids of *C. vulgaris* SAG 2602, including the omega-3 polyunsaturated fatty acids (PUFAs), hexadecatrienoic acid (HTA; 16:3), and alpha-linolenic acid (ALA; 18:3α). In contrast, in the tested strains of *Tetradesmus* (Chlorophyceae), which even represented the growth patterns with enhanced growth in high CO_2_, contents of the same fatty acids were lower and less gradable with N-limitation. It follows that also those strains for which our large-scale screening revealed just CO_2_ tolerance (growth pattern 5) might be interesting candidates for testing their exploitation in photobioreactors in high CO_2_ conditions.

While growth patterns with tolerance or even growth enhancement in high CO_2_ conditions prevailed in green algae, only one-third of the tested Stramenopiles algae strains exhibited growth pattern 5 and no patterns with enhanced growth. Stramenopiles growth, i.e., at least for the tested Xanthophyceae, Eustigmatophyceae, and Bacillariophyceae (diatoms) strains, appeared more sensitive towards higher CO_2_ concentrations, i.e., those algae mostly reacted with suppressed growth. This could be explained by stramenopile algae plastids originating from red algae. In all tested red algal strains, similar sensitivity, i.e., tolerance only to the 5% CO_2_ atmosphere or suppressed growth at all elevated CO_2_ levels (growth patterns 2 and 3) were found. Among stramenopile algae, the chances to find suitable candidates for sustainable exploitation in high CO_2_ conditions may be better among those strains originating from terrestrial habitats (e.g., *Vischeria* and *Heterococcus* strains). In contrast, the tested stramenopile algae from marine or brackish habitats, i.e., *Microchloropsis, Nannochloropsis,* and *Phaeodactylum*, had suppressed or even ceased growth under elevated CO_2_ atmospheres. These aspects will need to be tested further, focusing on a wider selection of stramenopile algae strains and high CO_2_ aerated liquid cultures.

Only a narrow range of green algal strains, representing the genera *Desmodesmus* and *Tetradesmus* of the order Sphaeropleales (Chlorophyceae), performed better under high CO_2_ atmospheres than pattern 5. Similar findings have been reported in previous studies [1, 27–29]. Members of Sphaeropleales, together with *Chlorella vulgaris* (Trebouxiophyceae), have been considered promising sources of biodiesel [1, 4, 14, 30, 31]. We regard our selection of defined test strains of the Chlorophyta as sufficiently diverse that other Chlorophyceae or Trebouxiophyceae genera with similar high performance could have been detected. However, this was not the case. We further examined 3 representatives of growth patterns 6 and 7 under high CO_2_ aeration in liquid cultures. The results recommend the new strains of *Tetradesmus obliquus* SAG 2607 and SAG 2608 and *T. bajacalifornicus* BIOTA 136 for producing biomass and carotenoids in high CO_2_ conditions. Those strains performed better in total carotenoid production than *Chlorella vulgaris*. Nitrogen limitation and even the combination of both, N-limitation with elevated CO_2_, clearly enhanced their production of fatty acids. This also recommends those strains for the sustainable production of fatty acids. The new green algal isolate, *Tetradesmus bajacalifornicus* BIOTA 136, was outstanding because it was the only test strain with enhanced growth under all tested levels (≥ 5%) of elevated CO_2_ atmospheres. While identifying most of the new test strains was straightforward by the ITS2 sequence comparisons, it was not for strain BIOTA 136. There were five closest reference strains representing *T. bajacalifornicus* [32] with sequence identities among them of 97–100%. The maximum identity of strain BIOTA 136 with one of them was 95%. Therefore, phylogenetic analyses and additional markers (e.g., [33]) are required further to investigate the species identity of strain BIOTA 136.

Remarkably, out of 9 top performers in our study, 5 were new strains isolated from terrestrial environments, such as from soil crusts of a semi-desert or other soil surfaces (*D. armatus* SAG 2635, *D. multivariabilis* SAG 2628, *T. bajacalifornicus* BIOTA 136, *T. obliquus* SAG 2607 and 2608). Those terrestrial habitats may be particularly interesting in recovering robust algal strains for sustainable exploitation in high CO_2_ conditions. Most of the tested green algal strains isolated from terrestrial habitats could tolerate CO_2_ levels beyond the relatively low threshold of 2–5%, considered as saturating for the CO_2_ uptake of the overwhelming majority of photoautotrophs [19]. Our study also shows that it is crucial to consider various isolates of the same species for optimizing algae exploitation. In the screening under elevated CO_2_ atmospheres on solid media, there were several cases with differences among various isolates of the same species (e.g., *Haematococcus pluvialis, Heterococcus viridis, Porphyridium purpureum*). Also, one of the two test strains, *Tetradesmus obliquus* strains SAG 2607 and SAG 2608, outperformed the other with slight differences in biomass productivity and the content of certain tested fatty acids under N-limitation in the high CO_2_ aerated liquid cultures.

The cyanobacteria strains used in our study represented the tested strains most sensitive to high CO_2_ levels. This corresponds to previous growth experiments with the cyanobacteria *Microcystis aeruginosa* and *Anabaena spiroides,* which showed inhibitory effects under elevated CO_2_ [32]. The reason for this inhibition remains unclear. However, by employing another genus diversity than those in previous works, half of the cyanobacteria strains used in our study exhibited tolerance toward the 5% CO2 atmosphere.

We employed algal growth on agar plates to expose 81 test strains to atmospheres of various elevated CO_2_ concentrations in the air. Plastic bags filled with atmospheres of different CO_2_ concentrations have been used to grow the algae in the liquid medium [26]. Although both methods appear suitable for a comprehensive screening, only the simple approach using agar plates allows for the extended duration of the cultivation because the nutrient supply may not become limiting within a short time [34]. The visual assessment of algal growth allowed us to assign the grades to the observed productivity. Comparisons with the control under ambient CO_2_ allowed for a robust estimation of the growth effects. The procedure was repeatedly tested by several independent investigators and resulted in convergent, stable assessments of the growth effects.

### Production of biomass, carotenoids, and fatty acids in 4 selected strains

All four selected strains produced increased amounts of biomass at 15% CO_2_. However, biomass alone is often not a sufficiently high value, particularly in competition with agriculture, where biomass production is more economical. Therefore, it was of interest to find compounds of added higher values. These are products for human nutrient supply and raw materials for pharmaceutics like fatty acids and pigments [35]. Algae as cell factories produce both types of compounds, but their content may frequently not be sufficiently high [36]. The supply of enhanced CO_2_ concentrations from inexpensive sources may increase the production of total lipids and carotenoids due to photosynthesis stimulation [37]. Algal biomass may also serve the production of biofuels [38, 39] and other high-value compounds [40, 41]. Due to the high CO_2_ supply, a more considerable increase in total fatty acids has been observed with the strain *Chlorella vulgaris* SAG 2606 (Fig. 4), while the three *Tetradesmus* strains exhibited higher carotenoid contents (Fig. 3).

To further enhance carotenoids and fatty acid contents, we manipulated the supply of nitrogen or phosphate [42, 43]. Nutrient deficiency, salt stress, and deficiency in trace elements are considered to trigger lipid accumulation [44, 45]. In the absence of nitrogen and with sufficient light, the cells continue to fix CO_2_. Still, they cannot synthesize proteins, which explains the accumulation of nitrogen-poor but carbon-rich storage substances such as lipids and starch [37, 46]. The high consumption of NADPH for fatty acid synthesis prevents an over-reduction of photosynthetic electron transport [47]. In addition, photo-oxidative stress is prevented, which can damage photosynthesis.

In all four selected test strains, reduction of the nitrogen concentration (conditions A-N and C-N) resulted in a significant increase in the total fatty acid content compared to that in complete nutrient solution under ambient CO_2_ (Fig. 4). However, a further increase in total fatty acid content due to the combination of N-deficiency with 15% CO_2_ in the air was only observed in *C. vulgaris* SAG 2606. Such modifications in the fatty acid profile between different phyla, classes, and genera could be anticipated and have been described even for species. However, that was mainly based on the different cultivation conditions [48]. CO_2_ supply up to 25% promoted cells of *T. bajacalifornicus* strain BBKLP-07 to produce high lipid contents [49]. These authors have not considered whether the stimulation of the lipid production can be further increased by nutrient starvation. In the three *Tetradesmus* strains tested in our study, N-depletion under elevated CO_2_ (condition C-N) caused a higher carotenoid synthesis (Fig. 3). The *Tetradesmus* strains may have a different strategy for dealing with oxidative stress than *Chlorella* in that the carotenoids detoxify the oxidative singlet oxygen [41]. The oxidative stress increases as photosynthesis is stimulated under 15% CO_2_, and additional N-deficiency supports the risk of photo-oxidation [50].

The supply of increased CO_2_ to the selected green alga strains strongly influenced their production of omega fatty acids. In our study, these were the 16:1 9z, 16:2, 16:3, 18:1 9z, 18:2 LA, 18:3α, and 18:4 fatty acids. When combined with nitrogen starvation, the total content of omega fatty acids increased compared to that in complete culture media under elevated CO_2_ conditions. The strain *C. vulgaris* SAG 2606 displayed a substantial effect in that respect (Fig. 4, top). Obviously, it is possible to stimulate the cells to convert the supply of high levels of CO_2_ into unsaturated fatty acids, especially under nitrogen limitations. A similar observation was reported by Ortiz Montoya and co-workers [47]. Fatty acid production increased beyond the well-known effect of nitrogen deficiency. The detailed analysis of fatty acids showed a relative increase in the 16:0 fatty acid, and the 16:3, and 18:3α polyunsaturated fatty acids (PUFAs) under nitrogen deficiency (Fig. 4; Additional file 3: Table S1). The ratio between the 16:3 and 18:3α fatty acids is considered important for the configuration and fluidity of the thylakoid membranes [51]. A high proportion of unsaturated fatty acids contributes to maintaining the fluidity, especially of the thylakoid membrane, since it has a very high proportion of membrane-integrated and -associated proteins [52]. The increased 16:0 fatty acid content accumulates in monogalactosyl diacylglycerol (MGDG), which indicates a rearrangement of the chloroplast membrane (for review, [19]). We also found an 18:1 9z fatty acid increase in *C. vulgaris* SAG 2606. It has also been reported for *Chlamydomonas,* where the authors correlate this result to the fast growth of algal cultures [53]. N-deprivation, in combination with increased CO_2,_ led to considerably enhanced content of the 18:1 9z fatty acid. The omega-9 fatty acid 18:1 9z is the most common fatty acid as a component of membrane lipids also in algae [54].

We also decreased the phosphate supply, which in general, led to similar results as nitrogen limitation. Still, the effects were much smaller than under nitrogen limitation. The network of regulation concerning nitrogen and phosphate metabolism affecting each other, especially in case of limitation, likely is the reason [55]. The cytosolic Ca^2+^ pool is affected and dampened by phosphate starvation but not by nitrogen starvation, as reported for *Arabidopis* roots [56]. In the diatom *Phaeodactylum tricornutum*, phospholipids and polyphosphates can serve as phosphate storage pools. These can be used during phosphate deficiency and delay the symptoms of phosphate starvation [57] compared to the fast occurrence of N-depletion symptoms [58].

## Conclusions

Growth patterns of a wide variety of defined microalgal strains from the SAG culture collection show tolerance and even growth enhancement when exposed to atmospheres of elevated CO_2_ concentrations. In particular, the isolates of green algae, Chlorophyceae and Trebouxiophyceae, from terrestrial habitats such as soil surfaces or temporary freshwater bodies exhibit enhanced productivity of carotenoids and fatty acids (including PUFAs) under elevated CO_2_ concentrations. This contrasts with cyanobacteria, unicellular red, and most stramenopile algae, whose growth is suppressed by elevated CO_2_ levels. In green algae of the Sphaeropleales (Chlorophyceae), i.e., *Tetradesmus* and *Desmodesmus*, and *Chlorella vulgaris* (Trebouxiophyceae), aeration with elevated CO_2_ into liquid culture not only increases their productivity in terms of biomass, but also the contents of carotenoids and total fatty acids, including omega-3 fatty acids. The contents of those valuable compounds can even be increased by macronutrient starvation, especially nitrogen. These findings recommend certain green algae originating from harsh terrestrial habitats for the economic and sustainable production of valuable compounds using inexpensive sources with high CO_2_ contents, such as flue and flaring gases.

## Methods

### New isolates, identification by sequence analyses

To search for productive and CO_2_ tolerant strains, 12 new strains of green algae were established (Table 1). Their origins included a range of harsh terrestrial environments, algal blooms on soil surfaces, and small shallow freshwater bodies of urban environments impaired by moderate pollution. Two novel strains, BIOTA 136 and BIOTA 153, isolated from biological soil crusts of arid climate regions in South Africa [59], were kindly provided by BIOTA, a long-term joint research project on biodiversity assessment (www.biota-africa.org). They are maintained at the SAG culture collection (Göttingen, Germany). For isolating the new algal strains, standard procedures as previously described [60, 61], were applied. For identification, the ITS2 rDNA regions of the new isolates were sequenced. Amplicons spanning from the 3′-end of 18S rRNA over the ITS1, 5.8S rRNA, and ITS2 regions to the 5′-end of the 26S rRNA gene were generated and sequenced as previously described [61, 62]. The ITS2 regions, about 238–244 base pairs long, were extracted from the obtained sequences using the *ITSx* software [63] in combination with own scripts. For species identification, the ITS2 sequences were queried on the portal of the NCBI Genbank database (https://www.ncbi.nlm.nih.gov/genbank/) using BLASTN [64]. The newly determined sequences are available from Genbank (Table 1).

### Growth experiments under elevated CO_2_ atmospheres with 81 algal strains

Out of the > 2500 defined algal strains available from the SAG culture collection, a pre-selection of 200 strains and one strain from the Culture Collection of Algae and Protozoa (CCAP; www.ccap.ac.uk), was performed. Those strains were re-examined for their growth on simple mineral media, as recorded over the many years of their maintenance. The same standard culture conditions in the temperature-controlled culture cabinets applied to perform the screening on solid media (see below) were used for all strains. All these strains were unicellular, capable of growing as cell suspensions. Finally, we compared the algal growth of the 81 strains. About half of them (36) are recorded as axenic in the catalog of the SAG culture collection. The test strains were maintained on solid (1.5% agar) culture media in Petri dishes (diameter about 6 cm) under the atmosphere of CO_2_ of various concentrations in air, i.e., enriched by CO_2_ gas (food grade; Linde, Munich, Germany) to 5, 15, and 25% CO_2_ to their controls under ambient CO_2_ concentration. The algal colonies, grown on the agar surface, were exposed to the CO_2_-enriched atmospheres with the petri dish lid closed but not sealed with parafilm. With agar plates, the duration of cultivation could be extended while the nutrient supply may not become limiting. The diffusion of CO_2_ into the agar could influence the pH of the growth media. However, considering the diffusion of CO_2_ from the enriched atmosphere is rather slow, the effect is not so great as to exceed the buffer capacity in the agar plates. For the growth on agar plates, standard culture media for the perpetual maintenance of the stock cultures, i.e., 3NBBM, BG11, ASM 15, and ASM 30, were used. For the composition of the growth media, see the website of the SAG culture collection (https://www.epsag.uni-goettingen.de, [65]). Algal colonies from the stock cultures mostly maintained on solid media in test tubes at the SAG culture collection were evenly distributed on agar plates using a Drigalski spatula. The strains were cultivated at light intensity of 40 μE s^− 1^ cm^− 2^, with a 14:10 day/night cycle. The temperature was 21 °C. Three plates (replicates) of each experimental condition were used, i.e., at elevated CO_2_ atmosphere, and the control at ambient CO_2_ in air was used (Fig. 1; Additional file 1: Fig. S1). Light- and temperature-controlled culture cabinets served for the growth tests. Experimental conditions and control were conducted in parallel and simultaneously in separated growth cabinets. The agar plates were randomly placed in the cabinets and their position was changed during the culturing several times to avoid biases due to a certain position in the cabinets. These were commercially available refrigerators with a glass front door (model FKvsl 3613, Liebherr, Ochsenhausen, Germany) inside which white LED fluorescent bulbs (Osram L 8 W/640 cool white) and pipes for atmosphere gassing were mounted. A microcomputer-controlled gassing system (QCAL Messtechnik GmbH, Munich, Germany) kept the CO_2_ level constant and monitored it during the growth experiments. A ventilator and arbitrary variation of the plates’ positions in the chamber circumvented possible imbalances of the CO_2_ concentration inside the growth chamber during an experiment. The experiments lasted about 2 weeks, i.e., 12–18 days.

### Growth experiments under direct gas bubbling with 4 selected algal strains

Four new isolates were cultured under conditions similar to those used for biotechnological applications. Here direct gas bubbling into the solution was used to increase the CO_2_ levels of liquid culture media. For liquid cultures, the “Kuhl medium”, i.e., the Kuhl and Lorenzen liquid culture medium [66], was used because it contained a higher buffering capacity. The bubbling air with 15% CO_2_ allowed a much better solubility and thus a possible pH shift. However, initial tests confirmed that pH does not interfere as a selection criterion in liquid culture due to the high buffer capacity of the medium (Fig. 2A). The focus of the experiments was on 15% CO_2_ in the air because this concentration is close to industrial exhaust fumes, which often reach 10–15% CO_2_ [67, 68]. Glass column photobioreactors (modified "Kniese" light thermostat, Hilke Feinmechanik GmbH, Uslar, Germany) of 4 cm in diameter and a volume of 400 ml to which a glass tube for gassing was attached were used [67]. Light from white LEDs dedicated to plant growth (sTube, Snaggi Lighting s.r.o., Prague, Czech Republic) at an intensity of 100 μE s^− 1^ cm^− 2^ and under a light/dark cycle of 14:10 h was applied. The glass columns were submerged in water baths kept at 25 °C. The cell density at the start of the experiments was 1.46 × 10^5^ per mL. The photobioreactors were gassed with various gas mixtures of CO_2_ in air, 0.1 L min^− 1^/tube (QCAL Messtechnik GmbH, Munich, Germany), i.e., from ambient (0.04%) to 25%. CO_2_. We tracked the pH by supplying 10 mM buffer systems (phosphate buffer or MOPS buffer) and daily pH control measurements. The changes in pH were small, and the available forms of carbon supply were HCO_3_^−^ and CO_3_^2−^ according to the pKs of 6.5 of the carbonate buffer system [22].

In addition to variation in CO_2_ supply, experiments under various nutrient supplies and combinations of both were performed. The cell density at the start of the experiment was the same as for the experiments with the complete growth medium (i.e., 1.46 × 10^5^ per mL). The nitrate concentration in the medium was decreased to only 5% of that of the Kuhl medium (condition “-N”). The decreased N-supply of 0.5 mM nitrate ensured that the cultures still grew well at the beginning of the experiment before they went into N-deficiency. To study the effect of phosphate deficiency, a phosphate-free nutrient solution was used. Because the pool of stored phosphate is large, a deficiency cannot be reached quickly. For experiments with phosphate deficiency (condition “-P”), the Kuhl medium phosphate buffer was replaced by MOPS buffer with the same ionic strength and buffering capacity following [69].

### Biomass, pigment production, and fatty acid levels of the four selected strains

After 1 week, the growth of the four selected algae strains cultivated in liquid media was determined via biomass production, measured as dry weight per mL suspension. The pigments, chlorophylls *a* and *b*, and total carotenoids were measured. To extract pigments, 2 mL of algal suspension were centrifuged (10 min, 1.8 × 10^4^ g). The pellet was re-suspended in 1 mL methanol/acetone (2:1) and incubated at 68 °C for 20 min. After removing the cell debris following centrifugation for 5 min at 1.8 × 10^4^ g, the absorptions (E, extinctions) were measured at 650 nm, 665 nm, and 473 nm with a Spectronic Genesys 20 (Thermo Fisher Scientific, Waltham MA, USA) photometer. Calculation of the pigment concentration [70] was as follows: chlorophyll *a*, 11.24 x E_665nm_ – 2.04 x E_650nm_ (μg mL^− 1^); chlorophyll *b*, 20.13 x E_650nm_ – 4.19 x E_665nm_ (μg mL^− 1^); carotenoids total: (1000 x E_473nm_ - 1.9 x chl. *a* - 63.14 x chl. *b*) / 214 (μg mL^− 1^). Also, total fatty acid levels were measured following the analyses of lipids as described previously [71, 72]. For the estimation of fatty acids as methyl esters (FAMEs), 1 ml of a methanolic solution containing 2.75% (v/v) H_2_SO_4_ (95–97%) and 2% (v/v) dimethoxypropane was added to 10 mg lyophilized algae culture. For later quantification of the fatty acids, 100 μg of triheptadecanoate was added as an internal standard, and the sample was incubated for 1 h at 80 °C. To extract the resulting FAMEs, 1.5 ml of saturated aqueous NaCl solution and 1.2 ml of hexane were added and centrifuged at 450 g for 10 min. The hexane phase was collected and dried under streaming nitrogen and redissolved in 0.1 ml acetonitrile. GC analysis was performed with an Agilent (Waldbronn, Germany) 6890 gas chromatograph fitted with a capillary DB-23 column (30 m × 0.25 mm; 0.25 μm coating thickness; J&W Scientific, Agilent, Waldbronn, Germany). Helium was used as carrier gas at a 1 ml/min flow rate. The temperature gradient was 150 °C for 1 min, 1–0 - 200 °C at 4 K min^− 1^, 200–250 °C at 5 K min^-1,^ and 250 °C for 6 min. Peak areas were collected with the ChemStation software (Agilent, Waldbronn, Germany).

### Statistical analysis

The growth experiments on solid media with CO_2_-enriched atmospheres in air were performed in triplicate, and those in liquid cultures with CO_2_ aeration in quadruplicate. Data were expressed as mean ± SD (standard deviation). Data were visualized using R version 4.1.3 [73], libraries from the tidy verse 1.3.1 package [74], and the ggplot2 package [75].

## Supplementary Information

**Additional file 1: Figure S1.** Examples for the visual assessment of algal growth on agar plates under atmospheres of elevated CO2 concentrations in air. Centre, photos of agar plates of four example growth experiments (lower row) and their corresponding controls (upper row), numbers are the assigned growth grades (see text). Next to the photos are the diagrams which show the mean growth from 3 replicate experiments in relation to controls under ambient CO2. Arrows mark examples for the different relations, i.e., 1, no change; >1, enhanced growth; <1 decreased growth; blue, example diagram for the new terrestrial green algal isolates (see Table 1); green, example diagram for the 4 strains selected for further testing (see text and Additional file 1: Figure S1).

**Additional file 2: Figure S2.** Diagrams showing the mean growth of the 81 tested algal strains on solid culture media under atmospheres of elevated CO2 concentrations in air in relation to controls under ambient CO2. Experiments were performed in triplicate. 1, no change; >1, enhanced growth; <1 decreased growth; blue diagrams, the 12 new terrestrial green algal isolates (Table 1); green, diagrams of the four strains selected for further testing (see text). Bold numbers, general patterns of mean growth (see text).

**Additional file 3: Table S1.** Detailed analysis of fatty acid (FAME) content [μmol/g dwt] of four selected green algal strains in the complete and nutrient-limited growth medium at ambient or 15% CO2 aeration in submerged culture. Experiments were performed in quadruplicate. AC, complete liquid medium aerated with ambient CO2; CC, complete medium and aeration at 15% CO2; A-P and C-P, aeration with ambient and 15% CO2 at phosphate limitation; A-N and C-N, aeration with ambient and 15% CO2 at nitrogen limitation.

## Data Availability

The datasets used and analysed during the current study are available from the corresponding author upon reasonable request.

## Abbreviations

CO_2_: Carbon dioxide
SAG: Culture collection of algae at Göttingen University, Germany
PUFAs: Polyunsaturated fatty acids
FAMEs: Fatty acid methyl esters
