# Supplementary material for: Testing for terrestrial and freshwater microalgae productivity under elevated CO2 conditions and nutrient limitation
Source: BMC Plant Biol. 2023 Jan 13;23:27. doi: 10.1186/s12870-023-04042-z (PMC9837994; doi:10.1186/s12870-023-04042-z)
Supplement: Supplementary file 1 — Additional file 1: Figure S1. Examples for the visual assessment of algal growth on agar plates under atmospheres of elevated CO2 concentrations in air. Centre, photos of agar plates of four example growth experiments (lower row) and their corresponding controls (upper row), numbers are the assigned growth grades (see text). Next to the photos are the diagrams which show the mean growth from 3 replicate experiments in relation to controls under ambient CO2. Arrows mark examples for the different relations, i.e., 1, no change; >1, enhanced growth; <1 decreased growth; blue, example diagram for the new terrestrial green algal isolates (see Table 1); green, example diagram for the 4 strains selected for further testing (see text and Additional file 1: Figure S1). [file 12870_2023_4042_MOESM1_ESM.pdf]

Supplementary Information

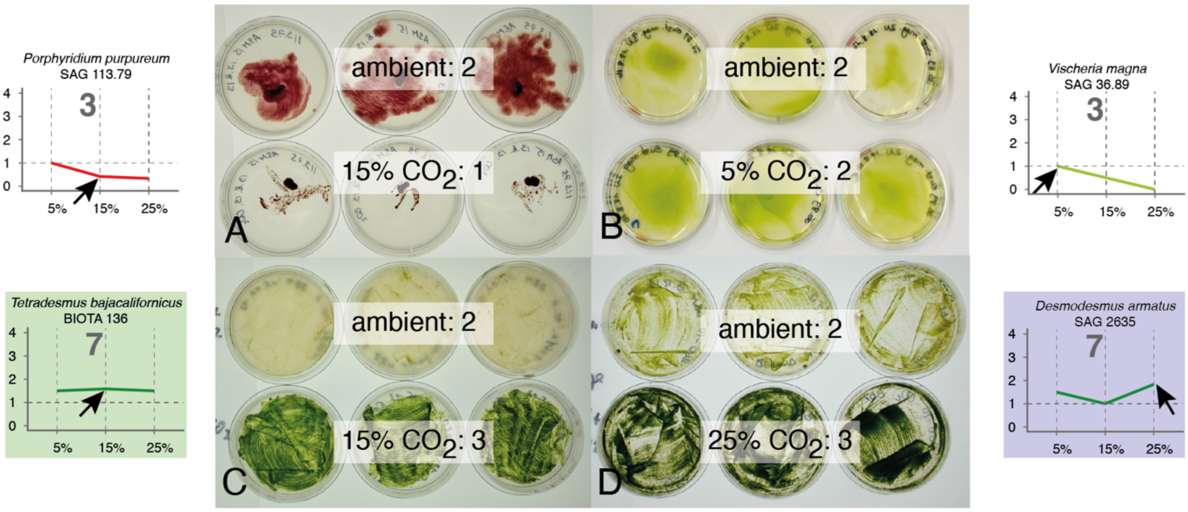

Additional file 1: Figure S1. Examples of the visual assessment of algal growth on agar plates under atmospheres of elevated CO_2 concentrations in air. Centre, photos of agar plates of four example growth experiments (lower row) and their corresponding controls (upper row), numbers are the assigned growth grades (see text). Next to the photos are the diagrams that show the mean growth from 3 replicate experiments in relation to controls under ambient CO_2. Arrows mark examples for the different relations, i.e., 1, no change; >1, enhanced growth; <1 decreased growth; blue, example diagram for the new terrestrial green algal isolates (see Table 1); green, example diagram for the four strains selected for further testing (see text and Additional file 1: Figure S1).
