# Supplementary material for: Testing for terrestrial and freshwater microalgae productivity under elevated CO2 conditions and nutrient limitation
Source: BMC Plant Biol. 2023 Jan 13;23:27. doi: 10.1186/s12870-023-04042-z (PMC9837994; doi:10.1186/s12870-023-04042-z)
Supplement: Supplementary file 2 — Additional file 2: Figure S2. Diagrams showing the mean growth of the 81 tested algal strains on solid culture media under atmospheres of elevated CO2 concentrations in air in relation to controls under ambient CO2. Experiments were performed in triplicate. 1, no change; >1, enhanced growth; <1 decreased growth; blue diagrams, the 12 new terrestrial green algal isolates (Table 1); green, diagrams of the four strains selected for further testing (see text). Bold numbers, general patterns of mean growth (see text). [file 12870_2023_4042_MOESM2_ESM.pdf]

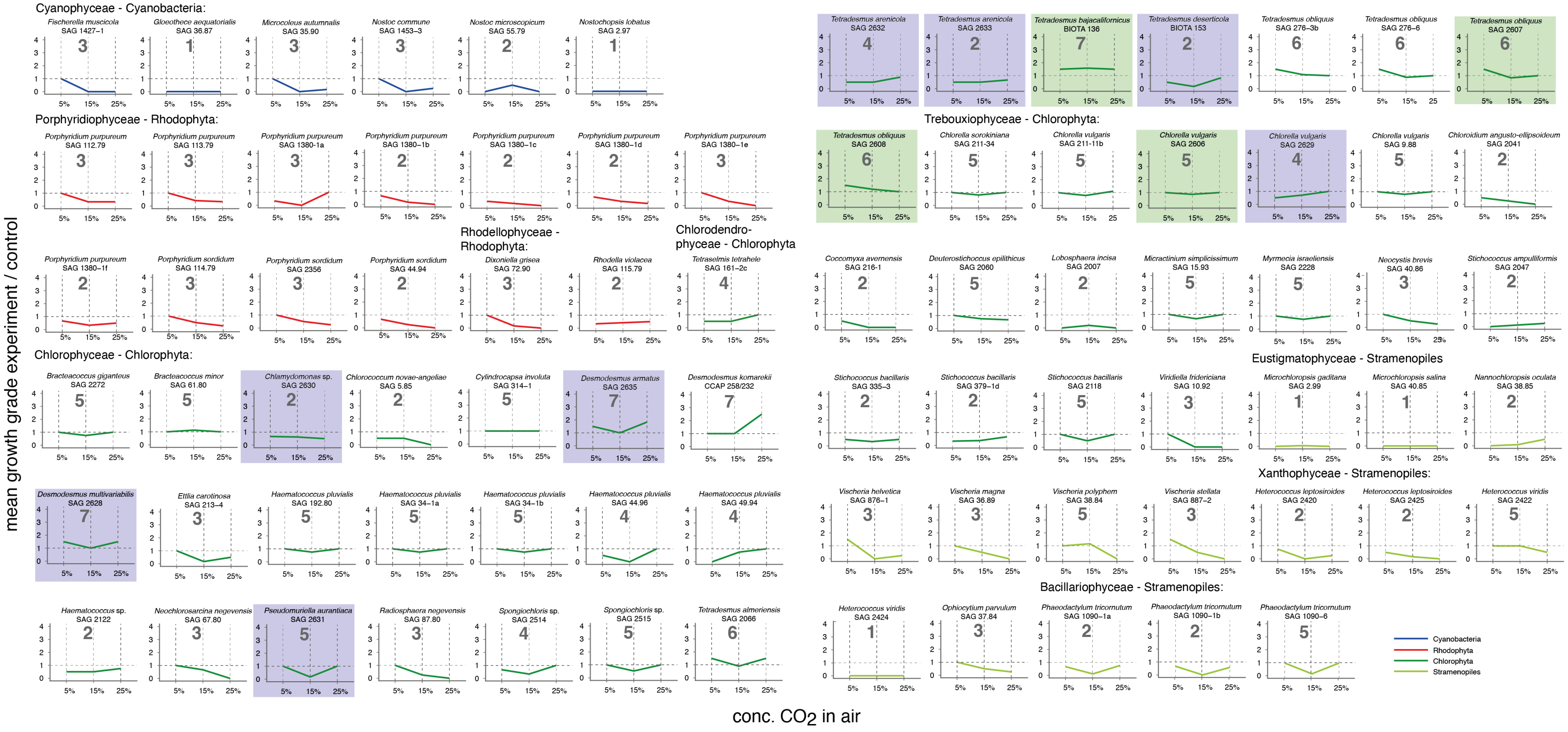
mean growth grade experiment / control
conc. CO_2 in air
Cyanophyceae - Cyanobacteria:
Fischerella muscicola SAG 1427–1 3
Gloeothece aequatorialis SAG 36.87 1
Microcoleus autumnalis SAG 35.90 3
Nostoc commune SAG 1453–3 3
Nostoc microscopicum SAG 55.79 2
Nostochopsis lobatus SAG 2.97 1
Porphyridiophyceae - Rhodophyta:
Porphyridium purpureum SAG 112.79 3
Porphyridium purpureum SAG 113.79 3
Porphyridium purpureum SAG 1380–1a 3
Porphyridium purpureum SAG 1380–1b 2
Porphyridium purpureum SAG 1380–1c 2
Porphyridium purpureum SAG 1380–1d 2
Porphyridium purpureum SAG 1380–1e 3
Porphyridium purpureum SAG 1380–1f 2
Porphyridium sordidum SAG 114.79 3
Porphyridium sordidum SAG 2356 3
Porphyridium sordidum SAG 44.94 2
Rhodellophyceae - Rhodophyta:
Dixoniella grisea SAG 72.90 3
Rhodella violacea SAG 115.79 2
Chlorodendrophyceae - Chlorophyta
Tetraselmis tetrahele SAG 161–2c 4
Chlorophyceae - Chlorophyta:
Bracteacoccus giganteus SAG 2272 5
Bracteacoccus minor SAG 61.80 5
Chlamydomonas sp. SAG 2630 2
Chlorococcum novae-angeliae SAG 5.85 2
Cylindrocapsa involuta SAG 314–1 5
Desmodesmus armatus SAG 2635 7
Desmodesmus komarekii CCAP 258/232 7
Desmodesmus multivariabilis SAG 2628 7
Ettlia carotinosa SAG 213–4 3
Haematococcus pluvialis SAG 192.80 5
Haematococcus pluvialis SAG 34–1a 5
Haematococcus pluvialis SAG 34–1b 5
Haematococcus pluvialis SAG 44.96 4
Haematococcus pluvialis SAG 49.94 4
Haematococcus sp. SAG 2122 2
Neochlorosarcina negevensis SAG 67.80 3
Pseudomuriella aurantiaca SAG 2631 5
Radiosphaera negevensis SAG 87.80 3
Spongiochloris sp. SAG 2514 4
Spongiochloris sp. SAG 2515 5
Tetradesmus almeriensis SAG 2066 6
Tetradesmus arenicola SAG 2632 4
Tetradesmus arenicola SAG 2633 2
Tetradesmus bajacalifornicus BIOTA 136 7
Tetradesmus deserticola BIOTA 153 2
Tetradesmus obliquus SAG 276–3b 6
Tetradesmus obliquus SAG 276–6 6
Tetradesmus obliquus SAG 2607 6
Tetradesmus obliquus SAG 2608 6
Trebouxiophyceae - Chlorophyta:
Chlorella sorokiniana SAG 211-34 5
Chlorella vulgaris SAG 211-11b 5
Chlorella vulgaris SAG 2606 5
Chlorella vulgaris SAG 2629 4
Chlorella vulgaris SAG 9.88 5
Chloroidium angusto-ellipsoideum SAG 2041 2
Coccomyxa avernensis SAG 216-1 2
Deuterostichococcus epilithicus SAG 2060 5
Lobosphaera incisa SAG 2007 2
Micractinium simplicissimum SAG 15.93 5
Myrmecia israeliensis SAG 2228 5
Neocystis brevis SAG 40.86 3
Stichococcus ampulliformis SAG 2047 2
Stichococcus bacillaris SAG 335–3 2
Stichococcus bacillaris SAG 379–1d 2
Stichococcus bacillaris SAG 2118 5
Viridiella fridericiana SAG 10.92 3
Eustigmatophyceae - Stramenopiles
Microchloropsis gaditana SAG 2.99 1
Microchloropsis salina SAG 40.85 1
Nannochloropsis oculata SAG 38.85 2
Xanthophyceae - Stramenopiles:
Vischeria helvetica SAG 876–1 3
Vischeria magna SAG 36.89 3
Vischeria polyphem SAG 38.84 5
Vischeria stellata SAG 887–2 3
Heterococcus leptosiroides SAG 2420 2
Heterococcus leptosiroides SAG 2425 2
Heterococcus viridis SAG 2422 5
Bacillariophyceae - Stramenopiles:
Heterococcus viridis SAG 2424 1
Ophiocytium parvulum SAG 37.84 3
Phaeodactylum tricornutum SAG 1090–1a 2
Phaeodactylum tricornutum SAG 1090–1b 2
Phaeodactylum tricornutum SAG 1090–6 5
5% 15% 25%
Cyanobacteria
Rhodophyta
Chlorophyta
Stramenopiles

Additional file 2: Figure S2. Diagrams showing the mean growth of the 81 tested algal strains on solid culture media under atmospheres of elevated CO_2 concentrations in air in relation to controls under ambient CO_2. Experiments were performed in triplicate. 1, no change; >1, enhanced growth; <1, decreased growth; blue, diagrams of new terrestrial green algal isolates (Table 1); green, diagrams of the four newly isolated strains selected for further testing (see text). Bold numbers, general patterns of mean growth (see text)
