# Supplementary material for: Testing for terrestrial and freshwater microalgae productivity under elevated CO2 conditions and nutrient limitation
Source: BMC Plant Biol. 2023 Jan 13;23:27. doi: 10.1186/s12870-023-04042-z (PMC9837994; doi:10.1186/s12870-023-04042-z)
Supplement: Supplementary file 3 — Additional file 3: Table S1. Detailed analysis of fatty acid (FAME) content [μmol/g dwt] of four selected green algal strains in the complete and nutrient-limited growth medium at ambient or 15% CO2 aeration in submerged culture. Experiments were performed in quadruplicate. AC, complete liquid medium aerated with ambient CO2; CC, complete medium and aeration at 15% CO2; A-P and C-P, aeration with ambient and 15% CO2 at phosphate limitation; A-N and C-N, aeration with ambient and 15% CO2 at nitrogen limitation. [file 12870_2023_4042_MOESM3_ESM.pdf]

Additional file 3: Table S1. Detailed analysis of fatty acid (FAME) content [µmol/g dwt] of four selected green algal strains in the complete and nutrient-limited growth medium at ambient or 15% CO_2 aeration in submerged culture. Experiments were performed in quadruplicate. AC, complete liquid medium aerated with ambient CO_2; CC, complete medium and aeration at 15% CO_2; A-P and C-P, aeration with ambient and 15% CO_2 at phosphate limitation; A-N and C-N, aeration with ambient and 15% CO_2 at nitrogen limitation.

Algal strain	culture medium	CO2 concentration	growth condition	total FAs	16:0	16:1,9z	16:2	16:3	16:4	18:0	18:1,9z	18:2.LA	18:3α	18:4
	complete	ambient	AC	262.6 ± 24.2	55.5 ± 8.4	6.6 ± 0.5	11.8 ± 2.1	46.3 ± 11.1	4.1 ± 0.2	0.6 ± 0.2	10.8 ± 1.3	23.6 ± 4.6	103.3 ± 19.1	---
		15%	CC	513.8 ± 22.6	101.1 ± 15.1	2.4 ± 1.9	29.8 ± 2.4	63.8 ± 9.7	4.2 ± 0.4	4.5 ± 0.8	99.1 ± 10.3	67.5 ± 17.1	141.4 ± 22.6	---
Chlorella vulgaris SAG 2606	- P	ambient	A-P	329.7 ± 14.7	66.1 ± 8.0	7.8 ± 0.8	11 ± 0.6	59.7 ± 7.1	4.2 ± 0.3	0.9 ± 0.3	20 ± 4.3	31.1 ± 1.9	129 ± 8.9	---
		15%	C-P	599.1 ± 35.6	113.9 ± 20.2	2.4 ± 0.9	34.8 ± 5.8	74.1 ± 8.1	4 ± 0.2	4.6 ± 0.7	130.1 ± 10.9	73.8 ± 15.9	161.4 ± 19.8	---
	- N	ambient	A-N	1158.4 ± 138.6	248.2 75.7	12.5 ± 3.1	25 ± 9.8	84.8 ± 24.1	---	30.3 ± 6.2	519.6 ± 102.4	81.7 ± 24.7	156.3 ± 40.7	---
		15%	C-N	1444.6 ± 72.0	351.3 ± 51.1	20.2 ± 2.3	24.3 ± 3.4	132 ± 10.8	---	18.1 ± 3.3	597.1 ± 40.1	87.1 ± 20.5	214.5 ± 20.1	---
	complete	ambient	AC	384.5 ± 26.4	91.7 ± 15.6	7.4 ± 3.0	20.7 ± 4.2	24.4 ± 5.3	6.7 ± 2.2	4.5 ± 1.1	89.8 ± 6.8	65.1 ± 10.7	62.1 ± 15.1	12.1 ± 2.4
		15%	CC	442.7 ± 42.2	104.2 ± 33	5.2 ± 1.9	5.5 ± 0.9	41.4 ± 9.3	28.4 ± 7.9	3.3 ± 0.8	70.8 ± 11.1	28.8 ± 5.3	132.1 ± 18.1	23 ± 8.1
Tetradesmus bajacalifornicus BIOTA136	- P	ambient	A-P	536.1 ± 63.4	131.8 ± 36.6	12.9 ± 3.1	24.6 ± 6.3	29.8 ± 7.0	4.8 ± 1.7	7.6 ± 2.1	179 ± 42.1	76.8 ± 18.1	56.4 ± 22.0	12.4 ± 1.9
		15%	C-P	409 ± 32.4	97.7 ± 19.1	3.9 ± 1.3	7.2 ± 1.8	36.2 ± 3.7	24.2 ± 6.7	3.2 ± 0.7	70.5 ± 13.0	28 ± 2.1	116.8 ± 20.1	21.3 ± 6.7
	- N	ambient	A-N	1059.3 ± 91.0	308.1 ± 33.5	2.9 ± 0.6	55.5 ± 17.8	23 ± 6.5	1.9 ± 0.6	23.7 ± 1.1	520 ± 78.4	119.3 ± 12.8	70 ± 22.1	14.6 ± 2.8
		15%	C-N	936.1 ± 43.3	284.9 ± 17.4	4.9 ± 1.8	11.8 ± 3.7	52.4 ± 21	17.4 ± 8.1	13.2 ± 2.1	315 ± 25.7	67.4 ± 4.3	131.8 ± 17.7	37.3 ± 7.3
		ambient	AC	270.6 ± 25.8	45.2 ± 5.1	8.8 ± 1.4	2.9 ± 0.7	9.2 ± 2.8	50.6 ± 12.1	0.77 ± 0.2	21.3 ± 2.3	27.1 ± 3.3	93.4 ± 21.6	11.3 ± 0.6
	complete	15%	CC	347.5 ± 23.5	77.6 ± 18.7	7.1 ± 1.7	3.2 ± 0.2	14 ± 1.9	38.5 ± 7.1	5.1 ± 1.1	89.1 ± 9.8	25.3 ± 3.9	74.4 ± 5.3	13.2 ± 2.1
Tetradesmus obliquus SAG 2607	- P	ambient	A-P	335.8 ± 29.1	59.8 ± 18.1	11.2 ± 2.8	4.1 ± 0.8	11.3 ± 2.2	56.6 ± 15.2	1.6 ± 0.3	49 ± 8.7	23.9 ± 3.1	106 ± 13.8	12.3 ± 1.1
		15%	C-P	450.1 ± 27.9	92.0 ± 19.0	7.5 ± 2.7	9.6 ± 1.6	17.8 ± 4.3	47.8 ± 8.1	7.9 ± 2.7	125 ± 12.7	33.2 ± 7.1	95.3 ± 9.8	14 ± 2.8
	- N	ambient	A-N	986.6 ± 115.5	236.1 ± 69.1	31 ± 10.2	29.3 ± 6.1	18.3 ± 6.1	25.2 ± 8.1	30.8 ± 7.1	447.4 ± 86.7	59.6 ± 17.7	90 ± 20.4	18.9 ± 3.9
		15%	C-N	896.1 ± 112.7	238.5 ± 50.1	6.1 ± 1.9	19.8 ± 4.3	26 ± 5.8	34.6 ± 9.8	16 ± 3.0	370.6 ± 50.1	46.4 ± 7.2	118.6 ± 5.5	19.5 ± 5.1
	complete	ambient	AC	292.5 ± 18.4	50.6 ± 7.2	11.7 ± 3.8	1.7 ± 0.4	5.1 ± 1.2	56.7 ± 1.6	0.4 ± 0.1	32.3 ± 10.6	28.4 ± 8.4	91.5 ± 8.4	14.1 ± 3.7
		15%	CC	381.9 ± 27.2	75.2 ± 15.1	9.3 ± 2.5	3.3 ± 0.1	16.2 ± 1.6	44.2 ± 8.6	5.3 ± 1.3	102.6 ± 15.1	28.9 ± 6.4	81.4 ± 12.1	15.5 ± 3.3
Tetradesmus obliquus SAG 2608	- P	ambient	A-P	356.4 ± 14.9	66.7 ± 4.8	10.2 ± 3.0	2.6 ± 0.9	7.7 ± 2.2	60.9 ± 2.2	1.1 ± 0.2	57.4 ± 10.5	26.3 ± 1.8	107 ± 8.0	16.5 ± 2.0
		15%	C-P	412.9 ± 29.7	86.8 ± 18.1	8.7 ± 3.6	3.0 ± 0.7	13.3 ± 3.8	44.3 ± 9.0	7.8 ± 2.0	120 ± 14.8	30.4 ± 6.1	82.7 ± 13.4	15.9 ± 2.4
	- N	ambient	A-N	1185.4 ± 81.7	243.4 ± 36.6	2.5 ± 0.7	15.5 ± 2.1	25.9 ± 3.6	38.4 ± 8.1	25.9 ± 2.6	564.2 ± 69.7	69.4 ± 15.8	103.6 ± 11.0	16.9 ± 4.2
		15%	C-N	766.8 ± 56.9	220.5 ± 44.1	7.1 ± 2.8	8.5 ± 1.1	24.7 ± 9.4	38.8 ± 8.5	11.6 ± 2.9	277.8 ± 28.1	46.7 ± 8.3	110.6 ± 15.7	20.5 ± 2.4
